# Differentiating vestibular migraine from Meniere's disease: an analysis of clinical features, videonystagmography, and caloric testing

**DOI:** 10.3389/fnins.2025.1745820

**Published:** 2026-01-13

**Authors:** Xueyan Zhang, Qirong Wang, Jiao Xu, Tao Zhou, Jin Xu, Zheng Liu, Xu Yang, Huaying Cai, Wencheng Luo, Mei Hu, Liying Chang

**Affiliations:** 1 Department of Neurology, Xiangyang Central Hospital, Affiliated Hospital of Hubei University of Arts and Science, Xiangyang, Hubei, China; 2 Department of Neurology, Peking University First Hospital, Beijing, China; 3 Department of Neurology, Sir Run Run Shaw Hospital, School of Medicine, Zhejiang University, Hangzhou, China; 4 Department of Neurology, Jingmen Duodao People's Hospital, Jingmen, Hubei, China

**Keywords:** caloric test, differential diagnosis, Meniere's disease, nystagmus, vestibular migraine

## Abstract

**Objective:**

Vestibular migraine (VM) and Meniere's disease (MD) are episodic vertigo disorders with overlapping symptoms, often leading to misdiagnosis. This study aimed to identify a cost-effective diagnostic method to distinguish VM and MD.

**Methods:**

In this retrospective study, 108 VM patients and 65 MD patients were enrolled. Clinical symptoms, interictal videonystagmography (VNG) findings, and caloric test results were analyzed and compared between the two groups.

**Results:**

The VM group had a significantly higher proportion of females (*p* < 0.001). No significant differences were observed in any features of interictal nystagmus, including spontaneous or positional nystagmus (all *p* > 0.05). However, the caloric test revealed a significantly higher proportion of canal paresis (CP) in MD patients compared to VM patients (*p* < 0.001).

**Conclusion:**

While clinical symptom profiles effectively distinguish VM from MD, interictal nystagmus analysis has limited diagnostic value. The caloric test is a reliable and practical tool, as the presence of significant canal paresis strongly indicates MD over VM. Combining symptom evaluation with caloric testing offers a cost-effective strategy for the differential diagnosis of these common vestibular disorders.

## 1 Introduction

Vestibular migraine (VM) and Meniere's disease (MD) are common episodic vertigo disorders. They share similar symptoms, including nausea, vomiting, photophobia, phonophobia, tinnitus, and subjective hearing loss (Villar-Martinez and Goadsby, 2024; Hoskin, 2022). These similar symptoms often lead to misdiagnosis, especially when some VM patients do not experience headache or when some MD patients are accompanied by headache (Chen et al., 2023). The treatment approaches for VM and MD differ greatly (Smyth et al., 2022; Basura et al., 2020). Therefore, it is essential to distinguish between the two conditions using cost-effective and straightforward methods.

Clinical studies aim to distinguish VM from MD, mainly through Gadolinium-contrasted magnetic resonance imaging (Gd-MRI) and vestibular testing (Chen et al., 2023). Current theories suggest that the pathogenesis of VM involves the activation and sensitization of the trigeminal vascular system, abnormal brain ion channels, and cortical spreading depression (Espinosa-Sanchez and Lopez-Escamez, 2015). Meanwhile, MD is primarily considered a peripheral vestibular disorder caused by endolymphatic hydrops (EH; Sajjadi and Paparella, 2008). Gd-MRI can visualize endolymphatic and peri-lymphatic structures in participants (Nakashima et al., 2007). In the Gd-MRI research, Nakada et al. (2014) identified that the key distinction between VM and MD is the presence of EH: 100% in MD patients vs. only 21% in VM patients. Other researchers found similar results; Wu et al. (2016) reported that EH was present in all definite MD patients. Gürkov et al. (2014) demonstrated that a few VM patients (21%) with auditory symptoms presented EH. Furthermore, Sun et al. (2017) indicated that the location of EH was different between VM and MD patients; MD patients exhibited cochlear and vestibular EH in 100% of cases, while VM patients showed suspected cochlear EH in 10% of cases, with no vestibular EH.

However, MRI scans are relatively expensive, whereas vestibular testing is more economical and straightforward. The videonystagmography (VNG) is a diagnostic tool that records involuntary eye movements using video-oculography to objectively assess the function of the vestibular and oculomotor systems by analyzing provoked, spontaneous, and positional nystagmus (Velenovsky, 2015). Young et al. (2019) reported that ictal spontaneous vertical nystagmus was highly specific for VM (93.0%), whereas horizontal nystagmus was specific for MD (82.1). Young et al. (2022) reported that all severe MD patients with spontaneous vertigo demonstrated ictal spontaneous nystagmus. But most studies have focused on the analysis of nystagmus during the ictal VM and MD. In our clinical practice, we observe that nystagmus also occurs in interictal periods. Furthermore, patients with VM and MD during the ictal period often experience intolerance to vestibular examinations. Therefore, it is essential to identify differences between VM and MD by examining nystagmus characteristics during interictal periods. The caloric test assesses unilateral vestibular hypofunction by irrigating the external auditory canal with warm and cold water or air to induce nystagmus, and the slow-phase velocity is used to determine canal paresis (CP; Perez and Rama-Lopez, 2003). The findings of caloric tests in VM and MD remain controversial. Most studies report a higher proportion of CP in MD patients than in VM patients (Sharon and Hullar, 2014; Taylor et al., 2012; Neff et al., 2012). In comparison, Martin-Sanz et al. (2014) did not observe any significant differences (*P* > 0.05) in the proportion of caloric test between the VM and MD population.

In our study, we explore potential differences between VM and MD by comparing their clinical features, interictal nystagmus, and caloric testing, thereby offering a foundation for clinical differential diagnosis.

## 2 Materials and methods

**Figure 1:**
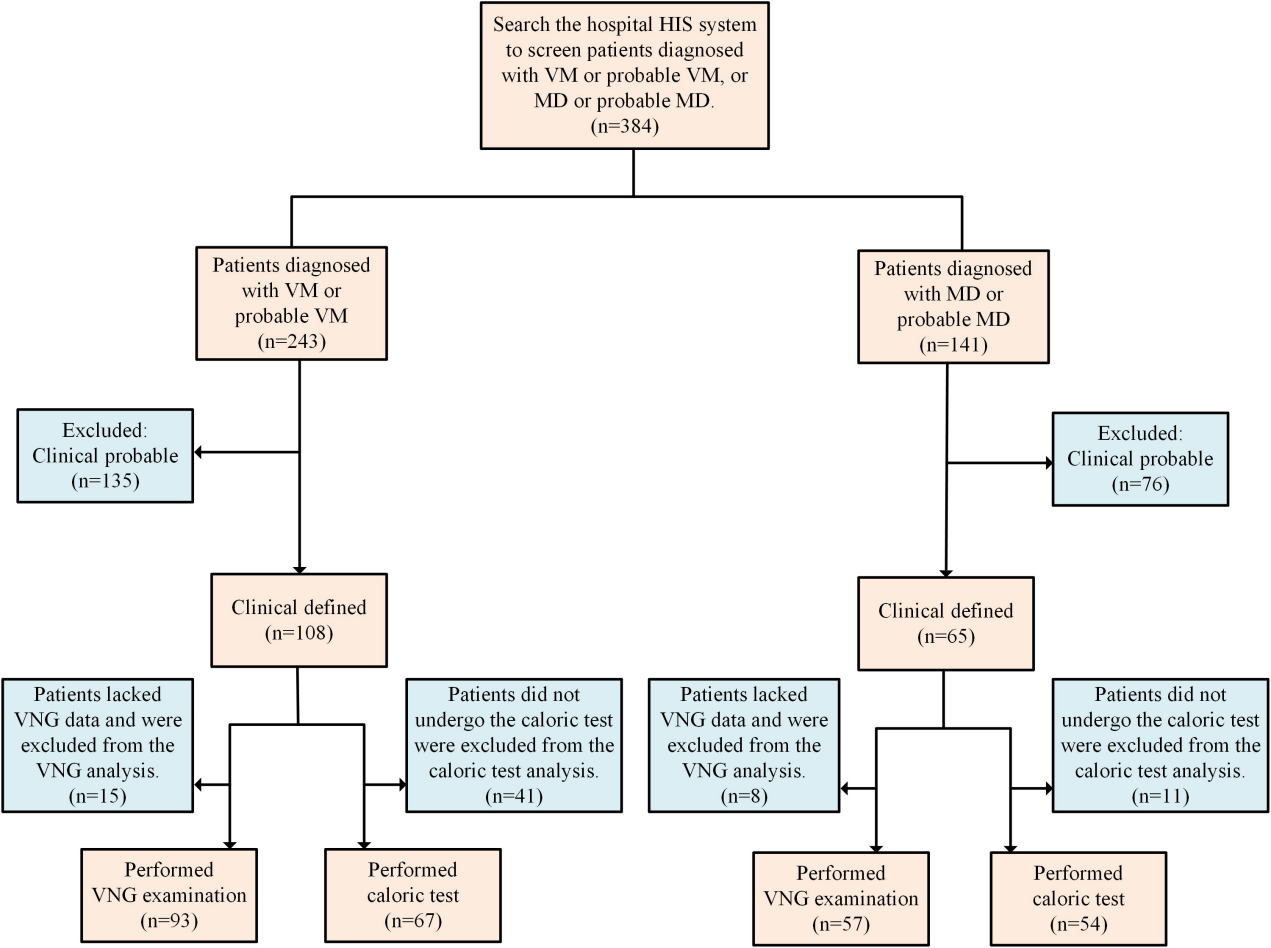
Flowchart of patients included. HIS, hospital information systems; VM, vestibular migraine; MD, Meniere's disease; VNG, videonystagmography.

In this retrospective study, we screened inpatient patients with VM, MD, and potential VM and MD from the hospital information systems (HIS) between January 2021 and September 2025. Diagnoses were clinically confirmed according to the VM diagnostic criteria of the International Classification of Headache Disorders, 3rd edition (ICHD-3; Arnold, 2018) and the MD diagnostic criteria of the Barany Society (Lopez-Escamez et al., 2015), based on medical records and clinical examinations (Figure 1). Patients with intracranial lesions confirmed by the magnetic resonance imaging (MRI) examination are excluded. The Ethics Committee of Xiangyang Central Hospital, Affiliated Hospital of Hubei University of Arts and Science, approved this study. All patients have written informed consent.

### 2.1 VNG examination procedures

The nystagmus was recorded with the VNG system, VertiGoggles (ZT-VNG-II, Shanghai ZEHNIT Medical Technology Co., Ltd., Shanghai, China). The patient lies supine with the head tilted 30 ° for a minimum recording period of 60 s for spontaneous nystagmus. In the supine roll test (SRT), the patient first lies on the exam table with the head tilted forward at 30 °. The head is then turned 90 ° to one side and maintained in this position for at least 60 s, even if no nystagmus is elicited. The patient then returns to the supine position. After a 60-s interval to avoid carry-over effects, the same procedure is repeated on the opposite side. In the Dix-Hallpike test (D-HT), the patient sits on the exam table with the head turned 45 ° to one side and eyes open, then leans back with the head hanging slightly (20–30 °) over the edge of the table. Nystagmus is recorded for at least 60 s in this position and maintained in this position for at least 60 s, even if no nystagmus is elicited. Following this, the patient returns to the sitting position. After a 60-s interval, the same procedure is performed on the other side. In the deep-head hanging test, patients lie back into a supine position with their head hanging at least 30 degrees below the horizontal plane and with a minimum recording period of 60 s (Bhattacharyya et al., 2017). While Benign Paroxysmal Positional Vertigo (BPPV) was not the focus of this study, performing the D-HT and SRT was for two reasons: first, to systematically exclude the presence of concurrent typical BPPV as part of a comprehensive vestibular assessment; and second, to characterize any provoked positional nystagmus, as a potential interictal finding in VM and MD patients. In addition to nystagmus direction, slow-phase velocity and the presence of torsional components were extracted from available VNG recordings for further interictal nystagmus characterization.

### 2.2 Caloric test procedures

The caloric test is performed with the patient in a supine position, head tilted forward (30 °) during bithermal water irrigations. First, cold water is used to irrigate the right and left ears. Then, warm water is used for the right and left ears. A 5-min interval is maintained between irrigations to prevent residual effects. Each irrigation lasts 30 s and uses 250 mL of water. The CP value is determined using the Jongkees formula (Jongkees et al., 1962), and a CP value of ≥25% is considered abnormal.

### 2.3 Statistical analysis

All statistical analyses are performed using SPSS (version 31.0, Chicago, IL, USA). Qualitative data are presented as numbers and percentages, and comparisons were made using the chi-square test or Fisher's exact test for 2 × 2 contingency tables. The normality of the quantitative data is assessed with the Shapiro-Wilk test. Data following a normal distribution are expressed as mean (standard deviation, SD), and differences are analyzed using the independent-samples *t*-test. For significant findings, effect sizes are reported as odds ratios (OR) with 95% confidence intervals (CI) for categorical variables and mean differences with 95% CI for continuous variables to indicate the magnitude of observed differences. Participants with missing data for a specific vestibular test were excluded only from the analyses pertaining to that test; no imputation methods were used. Two-tailed tests are used, and *p* < 0.05 is considered statistically significant.

## 3 Results

Finally, we include 108 patients with VM and 65 patients with MD. The proportion of female patients is higher in VM patients (85.2%) than in MD patients (46.2%; *p* < 0.001), with an odds ratio of 0.149 (95% CI: 0.072 to 0.307). There is no significant difference in age between the two groups (*p* = 0.153).

In the characteristics of dizziness, VM patients experience more vertigo and light-headed than MD patients (*p* = 0.045, with an odds ratio of 0.278 [95% CI: 0.078 to 0.995] and *p* = 0.019, with an odds ratio of 2.637 [95% CI: 1.203 to 5.782], respectively); there is no significant difference in rocking between the two groups (*p* = 0.103; Table 1).

**Table 1:** Demographic information and clinical characteristics for patients with VM and MD.

**Variable**	**VM (*n* = 108)**	**MD (*n* = 65)**	***p*-value**
Gender, male, *n* (%)	16 (14.8)	35 (53.8)	< 0.001^***^ 0.149 (0.072, 0.307)
Age, mean (SD), years	63.4 (12.7)	60.6 (12.9)	0.153
History of headache, *n* (%)	56 (51.9)	5 (7.7)	< 0.001^***^ 12.923 (4.815, 34.686)
**Characteristics of dizziness**
Vertigo, *n* (%)	92 (85.2)	62 (95.4)	0.045^*^ 0.278 (0.078, 0.995)
Rocking, *n* (%)	34 (31.5)	29 (44.6)	0.103
Light-headed, *n* (%)	35 (32.4)	10 (15.4)	0.019^*^ 2.637 (1.203, 5.782)

Comparisons were made using Fisher's exact test for binary variables or the chi-square test as appropriate. For significant comparisons (*p* < 0.05), the odds ratio (95% confidence interval) is provided in parentheses.

^*^p < 0.05; ^***^p < 0.001.

VM, vestibular migraine; MD, Meniere's disease; SD, standard deviation; n, number.

**Figure 2:**
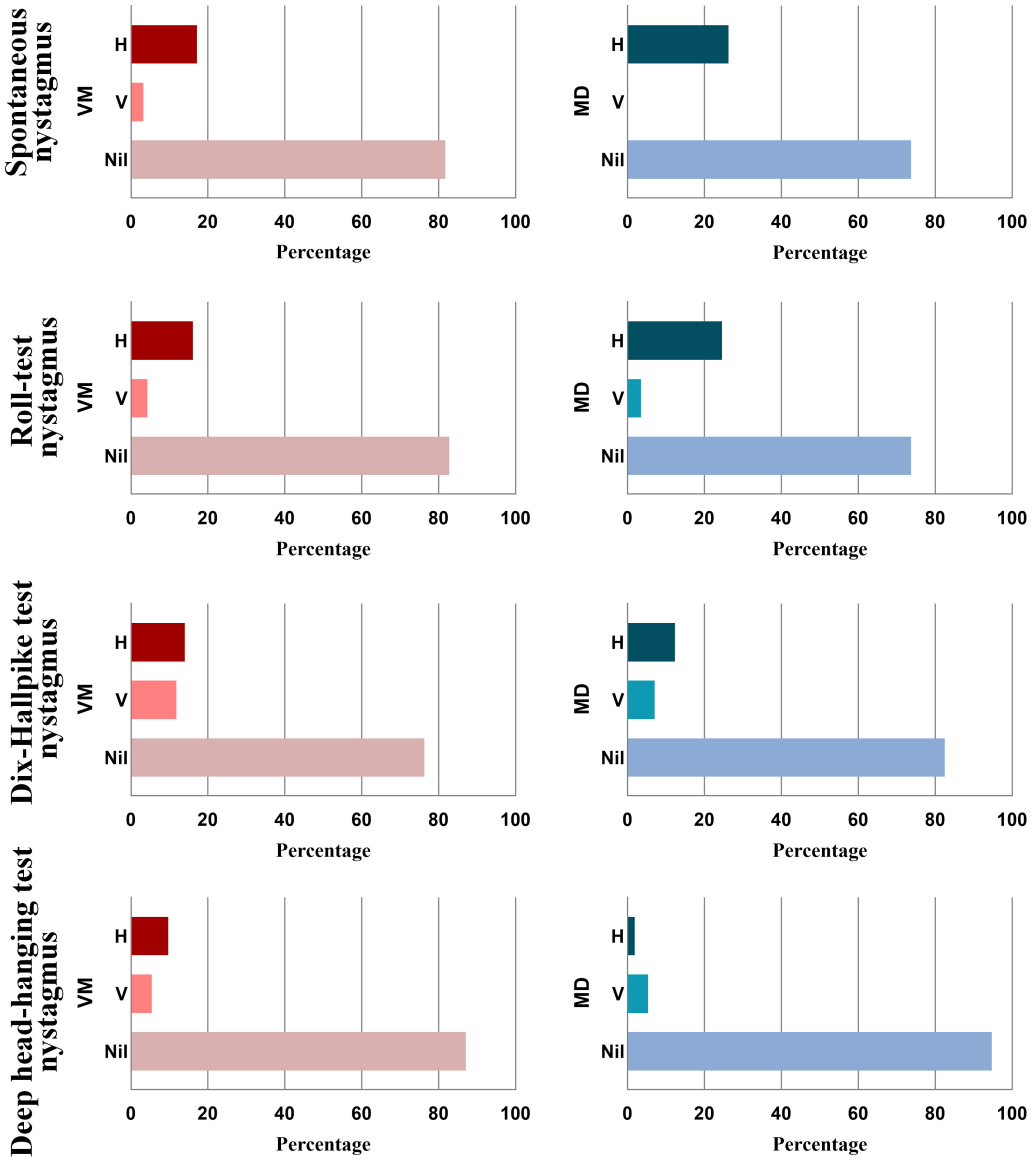
Comparison of spontaneous nystagmus, nystagmus of Roll-test, Dix-Hallpike test, and deep head-hanging test, and nil nystagmus in VM and MD patients. VM, vestibular migraine; MD, Meniere's disease; H, horizontal; V, vertical.

We find no significant difference in nystagmus characteristics, including spontaneous nystagmus, positional nystagmus (Roll-test, Dix-Hallpike test, and deep head-hanging test), and the proportion of nystagmus direction change or nil nystagmus (all *p* > 0.05). Fifteen VM patients and eight MD patients lacked VNG examination data are excluded (Table 2 and Figure 2). In addition to nystagmus direction, quantitative and qualitative parameters of interictal nystagmus were further analyzed, including slow-phase velocity (SPV) and the presence of torsional components. No significant differences were observed between the VM and MD groups in SPV values for spontaneous or positional nystagmus (all *p* > 0.05; Table 3). Torsional components were infrequently observed and, when present, occurred with similar frequency in both groups (all *p* > 0.05; Table 3).

**Table 2:** Nystagmus characteristics of patients with VM and MD.

**Nystagmus characteristic**	**VM (*n* = 93)^#^**	**MD (*n* = 57)^#^**	***p*-value**
Spontaneous nystagmus, *n* (%)	17 (18.3)	15 (26.3)	0.244
Horizontal, *n* (%)	16 (17.2)	15 (26.3)	0.181
Vertical, *n* (%)	3 (3.2)	0 (0)	0.171
Up beating nystagmus, *n* (%)	0 (0)	0 (0)	NA
Down beating nystagmus, *n* (%)	3 (3.2)	0 (0)	0.171
Positional nystagmus (Supine Roll-test), *n* (%)	16 (17.2)	15 (26.3)	0.181
Horizontal, *n* (%)	15 (16.1)	14 (24.6)	0.204
Vertical, *n* (%)	4 (4.3)	2 (3.5)	0.810
Up beating nystagmus, *n* (%)	0 (0)	0 (0)	NA
Down beating nystagmus, *n* (%)	4 (4.3)	2 (3.5)	0.810
Positional nystagmus (Dix-Hallpike test), *n* (%)	22 (23.7)	10 (17.5)	0.375
Horizontal, *n* (%)	13 (14.0)	7 (12.3)	0.767
Vertical, *n* (%)	11 (11.8)	4 (7.0)	0.340
Up beating nystagmus, *n* (%)	8 (8.6)	1 (1.8)	0.087
Down beating nystagmus, *n* (%)	9 (9.7)	3 (5.3)	0.333
Positional nystagmus (Deep head-hanging test), *n* (%)	12 (12.9)	3 (5.3)	0.130
Horizontal, *n* (%)	9 (9.7)	1 (1.8)	0.059
Vertical, *n* (%)	5 (5.4)	3 (5.3)	0.976
Up beating nystagmus, *n* (%)	1 (1.1)	1 (1.8)	0.725
Down beating nystagmus, *n* (%)	4 (4.3)	2 (3.5)	0.810
Nystagmus direction change, *n* (%)	11 (11.8)	4 (7.0)	0.340
Nil nystagmus, *n* (%)	59 (63.4)	30 (52.6)	0.191

^#^Fifteen VM patients and eight MD patients lacked electronystagmography examination data.

VM, vestibular migraine; MD, Meniere's disease; n, number; NA, not applicable.

**Table 3:** Nystagmus characteristics in VM and MD patients: slow-phase velocity and torsional components.^※^

**Test condition**	**Parameter**	**VM (*n* = 108)**	**MD (*n* = 65)**	***p*-value**
Spontaneous nystagmus	SPV (°/s)^#^	3.1 (2.4)	3.0 (2.0, 4.0)	0.303
	Torsional component, *n* (%)	0 (0)	0 (0)	NA
SRT nystagmus	SPV (°/s)	4.0 (2.4)	4.9 (3.6)	0.436
	Torsional component, *n* (%)	1 (0.9)	1 (1.5)	0.715
D-HT nystagmus	SPV (°/s)	5.0 (3.0, 11.3)	3.0 (2.0, 4.0)	0.053
	Torsional component, *n* (%)	11 (10.2)	3 (4.6)	0.193
Deep head-hanging test nystagmus	SPV (°/s)	2.0 (2.0, 9.8)	2.0 (2.0, 2.0)	0.328
	Torsional component, *n* (%)	3 (2.8)	2 (3.1)	0.909

^※^In a positional test where multiple nystagmus occurrences are observed, we take the average to calculate the SPV for that positional test.

^#^Normally distributed data are expressed as mean (SD), and comparisons between two groups are performed using independent samples *t*-tests; non-normally distributed data are expressed as median (Q1, Q3), and comparisons between two groups are performed using non-parametric tests.

VM, vestibular migraine; MD, Meniere's disease; SRT, Supine Roll-test; D-HT, Dix-Hallpike test; SPV, slow-phase velocity; NA, not applicable; n, number; SD, standard deviation; Q1, First quartile; Q3, Third Quartile.

**Figure 3:**
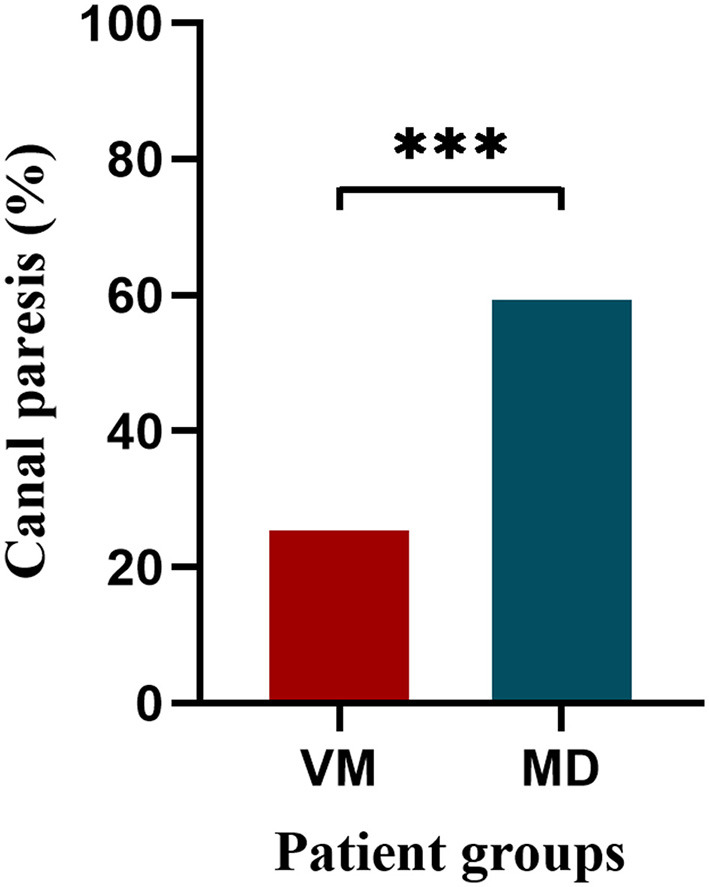
Comparison of canal paresis in VM and MD patients. VM, vestibular migraine; MD, Meniere's disease. ***p < 0.001.

In the caloric test, the proportion of CP is higher in MD patients than in VM patients (*p* < 0.001). Fourteen VM patients were excluded: they lacked caloric test data; 21 patients refused the examination due to intolerance to the caloric stimulation (primarily due to provocation of severe vertigo or nausea); and six patients did not undergo the test due to cerumen impaction or tympanic membrane perforation. Nine MD patients lacked caloric test data, and two patients did not undergo the test due to cerumen impaction or tympanic membrane perforation are excluded (Table 4 and Figure 3).

**Table 4:** Caloric test of patients with VM and MD.

**Caloric test**	**VM (*n* = 67)**	**MD (*n* = 54)**	***p*-value**
CP, *n* (%)	17 (25.4)	32 (59.3)	< 0.001^***^

^※^Among the 108 patients with vestibular migraine, 67 patients underwent the caloric test, 14 patients lacked caloric test data, 21 patients refused the examination due to intolerance to the caloric stimulation (primarily due to provocation of severe vertigo or nausea), and 6 patients did not undergo the test due to cerumen impaction or tympanic membrane perforation. Separately, among 65 patients with Meniere's Disease, 54 patients underwent the caloric test, 9 patients lacked caloric test data, and 2 patients did not undergo the test due to cerumen impaction or tympanic membrane perforation. ^***^p < 0.001. VM, vestibular migraine; MD, Meniere's disease; CP, canal paresis; n, number.

## 4 Discussions

This study compared the clinical features, interictal VNG findings, and caloric testing results between patients with VM and MD. Our findings revealed that VM was more common in females. Although no significant differences in interictal nystagmus characteristics were observed between the two groups, the proportion of CP in the caloric test was significantly higher in MD than in VM. These results suggest that caloric testing may be a reliable and cost-effective method for distinguishing MD from VM in clinical practice.

Our findings both support and challenge existing research on VM and MD differentiation. The demographic and symptomatic profiles we identified closely align with previous studies. The female predominance in VM (Beh, 2022).

Our study found no significant differences in interictal nystagmus between VM and MD. Notably, a subset of patients showed transient or direction-changing positional nystagmus during tests such as the Roll test, Dix–Hallpike maneuver, or deep head-hanging test, with no apparent diagnostic specificity. The presence of such variable nystagmus patterns further supports the idea that both disorders may involve central adaptive mechanisms. In VM, transient dysfunction within vestibular nuclei and cerebellar pathways may cause mixed-pattern eye movements, as previously reported in studies of vestibulo-cerebellar modulation (Noseda, 2022; Harno et al., 2003; Zhai et al., 2025). In MD, fluctuating endolymphatic hydrops could lead to intermittent changes in labyrinthine input, resulting in dynamic central reweighting of vestibular signals (Bance et al., 1991; Nakashima et al., 2016). These overlapping pathophysiological processes could explain the nonspecific interictal nystagmus patterns seen in our study, aligning with emerging evidence that both peripheral and central mechanisms influence nystagmus phenotypes in these disorders (Pokhrel et al., 2025; Li et al., 2022; Bednarczuk et al., 2019; Lee et al., 2022, 2023).

By incorporating SPV and torsional components into the analysis of interictal nystagmus, our study provides a more comprehensive assessment of eye movement characteristics in VM and MD. Despite this expanded evaluation, no diagnostically meaningful differences were identified between the two conditions. This suggests that interictal nystagmus in both VM and MD likely reflects transient or compensated vestibular dysfunction rather than fixed peripheral or central lesions. In VM, fluctuating central vestibular network excitability may result in low-amplitude, variable nystagmus patterns, while in MD, intermittent alterations in labyrinthine input caused by endolymphatic hydrops may be partially compensated during interictal phases.

The caloric test results further support the existence of distinct vestibular mechanisms underlying VM and MD. MD is widely recognized as a peripheral disorder characterized by endolymphatic hydrops, which disrupts labyrinthine mechanics and causes asymmetric canal function (Mohseni-Dargah et al., 2023; Park et al., 2021). In contrast, VM is believed to result from temporary dysfunction within central vestibular networks, potentially mediated by trigeminovascular activation, cortical spreading depression, and abnormal ion channel activity (Smyth et al., 2022). These mechanisms of VM can produce vestibular symptoms without causing structural damage to the peripheral system, which explains why caloric asymmetry is rarely observed in VM.

From a clinical standpoint, these findings underscore the importance of integrating symptom profiles with caloric testing to improve diagnostic accuracy. A CP of ≥25% highly suggests MD, while normal caloric responses, especially in patients exhibiting migraine symptoms such as photophobia or phonophobia, more strongly suggest VM. However, it is important to note that a normal caloric test result does not rule out MD, as unilateral vestibular hypofunction may not be detectable, particularly in the early stages of the disease. Given the high cost and limited availability of gadolinium-enhanced MRI for visualizing endolymphatic hydrops, caloric testing remains a practical and valuable tool for differential diagnosis. Incorporating these insights into clinical evaluation can support more targeted treatment approaches.

### 4.1 Study limitations

Several limitations should be acknowledged. First, the retrospective design and single-center setting of the study may introduce selection bias. Second, the unavailability of video head impulse test (v-HIT) data represents a notable constraint, as the combination of caloric testing (assessing low-frequency function) and v-HIT (assessing high-frequency function) may offer greater diagnostic specificity, as suggested by recent literature (Chen et al., 2023; Mavrodiev et al., 2024). Third, as noted in the results, a higher proportion (38%) of VM patients were excluded from the final caloric test analysis compared to MD patients (17%). A *post-hoc* comparison revealed that excluded VM patients were older, and excluded MD patients more frequently reported a history of headache (Supplementary Table 1). However, the key differentiating clinical features, such as tinnitus and hearing loss in MD, and photophobia/phonophobia in VM, did not differ significantly between the included and excluded subgroups within each diagnosis. Nevertheless, this differential attrition represents a potential source of selection bias. The primary reason for exclusion in the VM group was intolerance to the caloric test, which may reflect the heightened vestibular sensitivity characteristic of the disorder. Finally, Fixation suppression testing was performed only in a subset of patients and was therefore not included in the comparative analysis, which may have limited the ability to further distinguish central from peripheral nystagmus patterns. Future prospective studies incorporating multimodal vestibular assessments are necessary to reinforce these findings.

## 5 Conclusions

In conclusion, characteristics of interictal nystagmus have limited diagnostic value, but caloric asymmetry is a strong indicator for differentiating the two conditions. Cost-effective vestibular function tests combined with symptom profiling may improve the diagnosis and management of VM and MD.

## Data Availability

The raw data supporting the conclusions of this article will be made available by the authors, without undue reservation.

## References

[B1] Arnold M. (2018). Headache classification committee of the international headache society (IHS) the international classification of headache disorders, 3rd edition. Cephalalgia 38, 1–211. doi: 10.1177/0333102417738202 29368949

[B2] Bance M. Mai M. Tomlinson D. Rutka J. (1991). The changing direction of nystagmus in acute Menière's disease: pathophysiological implications. Laryngoscope 101, 197–201. doi: 10.1288/00005537-199102000-00017 1992273

[B3] Basura G. J. Adams M. E. Monfared A. Schwartz S. R. Antonelli P. J. Burkard R. . (2020). Clinical practice guideline: Ménière's disease. Otolaryngol. Head Neck Surg. 162, S1–S55. doi: 10.1177/0194599820909438 32267799

[B4] Bednarczuk N. F. Bonsu A. Ortega M. C. Fluri A. S. Chan J. Rust H. . (2019). Abnormal visuo-vestibular interactions in vestibular migraine: a cross sectional study. Brain 142, 606–616. doi: 10.1093/brain/awy355 30759189 PMC6391603

[B5] Beh S. C. (2022). Vestibular migraine. Curr. Neurol. Neurosci. Rep. 22, 601–609. doi: 10.1007/s11910-022-01222-6 36044103

[B6] Bhattacharyya N. Gubbels S. P. Schwartz S. R. Edlow J. A. El-Kashlan H. Fife T. . (2017). Clinical practice guideline: benign paroxysmal positional vertigo (update). Otolaryngol. Head Neck Surg. 156(3_suppl), S1–S47. doi: 10.1177/0194599816689667 28248609

[B7] Chen J. Y. Guo Z. Q. Wang J. Liu D. Tian E. Guo J. Q. . (2023). Vestibular migraine or Meniere's disease: a diagnostic dilemma. J. Neurol. 270, 1955–1968. doi: 10.1007/s00415-022-11532-x 36562849 PMC10025214

[B8] Espinosa-Sanchez J. M. Lopez-Escamez J. A. (2015). New insights into pathophysiology of vestibular migraine. Front. Neurol. 6:12. doi: 10.3389/fneur.2015.00012 25705201 PMC4319397

[B9] Gürkov R. Kantner C. Strupp M. Flatz W. Krause E. Ertl-Wagner B. . (2014). Endolymphatic hydrops in patients with vestibular migraine and auditory symptoms. Eur. Arch. Otorhinolaryngol. 271, 2661–2667. doi: 10.1007/s00405-013-2751-2 24121780

[B10] Harno H. Hirvonen T. Kaunisto M. A. Aalto H. Levo H. Isotalo E. . (2003). Subclinical vestibulocerebellar dysfunction in migraine with and without aura. Neurology 61, 1748–1752. doi: 10.1212/01.WNL.0000098882.82690.65 14694041

[B11] Hoskin J. L. (2022). Ménière's disease: new guidelines, subtypes, imaging, and more. Curr. Opin. Neurol. 35, 90–97. doi: 10.1097/WCO.0000000000001021 34864755

[B12] Jongkees L. B. Maas J. P. Philipszoon A. J. (1962). Clinical nystagmography. A detailed study of electro-nystagmography in 341 patients with vertigo. Pract. Otorhinolaryngol. 24, 65–93. doi: 10.1159/000274383 14452374

[B13] Lee J. Park J. Y. Shin J. E. Kim C. H. (2023). Direction-changing spontaneous nystagmus in patients with dizziness. Eur. Arch. Otorhinolaryngol. 280, 2725–2733. doi: 10.1007/s00405-022-07761-5 36454383

[B14] Lee S. U. Kim H. J. Choi J. Y. Kim B. J. Kim J. S. (2022). Discordant horizontal-torsional nystagmus: a sign of posterior semicircular canal dysfunction. J. Neurol. 269, 5038–5046. doi: 10.1007/s00415-022-11155-2 35543743

[B15] Li Z. Y. Si L. H. Shen B. Yang X. (2022). Altered brain network functional connectivity patterns in patients with vestibular migraine diagnosed according to the diagnostic criteria of the Bárány Society and the International Headache Society. J. Neurol. 269, 3026–3036. doi: 10.1007/s00415-021-10868-0 34792633 PMC9119883

[B16] Lopez-Escamez J. A. Carey J. Chung W. H. Goebel J. A. Magnusson M. Mandalà M. . (2015). Diagnostic criteria for Menière's disease. J. Vestib. Res. 25, 1–7. doi: 10.3233/VES-150549 25882471

[B17] Martin-Sanz E. Vargas Salamanca E. Marqués Cabrero A. Esteban J. Muerte I. Sanz-Fernández R. . (2014). Value of clinical data and vestibular testing in a population of 101 patients with recurrent vestibulopathy. Clin. Otolaryngol. 39, 311–315. doi: 10.1111/coa.12287 25042894

[B18] Mavrodiev V. Strupp M. Vinck A. S. van de Berg R. Lehner L. (2024). The dissociation between pathological caloric testing and a normal video head impulse test helps differentiate between Menière's disease, vestibular migraine, and other vestibular disorders: a confirmatory study in a large cohort of 2,101 patients. Front. Neurol. 15:1449261. doi: 10.3389/fneur.2024.1449261 39206283 PMC11350975

[B19] Mohseni-Dargah M. Falahati Z. Pastras C. Khajeh K. Mukherjee P. Razmjou A. . (2023). Meniere's disease: pathogenesis, treatments, and emerging approaches for an idiopathic bioenvironmental disorder. Environ. Res. 238(Pt 1):116972. doi: 10.1016/j.envres.2023.116972 37648189

[B20] Nakada T. Yoshida T. Suga K. Kato M. Otake H. Kato K. . (2014). Endolymphatic space size in patients with vestibular migraine and Ménière's disease. J. Neurol. 261, 2079–2084. doi: 10.1007/s00415-014-7458-9 25099513

[B21] Nakashima T. Naganawa S. Sugiura M. Teranishi M. Sone M. Hayashi H. . (2007). Visualization of endolymphatic hydrops in patients with Meniere's disease. Laryngoscope 117, 415–420. doi: 10.1097/MLG.0b013e31802c300c 17279053

[B22] Nakashima T. Pyykkö I. Arroll M. A. Casselbrant M. L. Foster C. A. Manzoor N. F. . (2016). Meniere's disease. Nat. Rev. Dis. Prim. 2:16028. doi: 10.1038/nrdp.2016.28 27170253

[B23] Neff B. A. Staab J. P. Eggers S. D. Carlson M. L. Schmitt W. R. Van Abel K. M. . (2012). Auditory and vestibular symptoms and chronic subjective dizziness in patients with Ménière's disease, vestibular migraine, and Ménière's disease with concomitant vestibular migraine. Otol. Neurotol. 33, 1235–1244. doi: 10.1097/MAO.0b013e31825d644a 22801040

[B24] Noseda R. (2022). Cerebro-cerebellar networks in migraine symptoms and headache. Front. Pain Res. 3:940923. doi: 10.3389/fpain.2022.940923 35910262 PMC9326053

[B25] Park C. J. Cho Y. S. Chung M. J. Kim Y. K. Kim H. J. Kim K. . (2021). A fully automated analytic system for measuring endolymphatic hydrops ratios in patients with Ménière disease via magnetic resonance imaging: deep learning model development study. J. Med. Internet Res. 23:e29678. doi: 10.2196/29678 34546181 PMC8493456

[B26] Perez N. Rama-Lopez J. (2003). Head-impulse and caloric tests in patients with dizziness. Otol. Neurotol. 24, 913–917. doi: 10.1097/00129492-200311000-00016 14600474

[B27] Pokhrel P. K. Hall R. Pendergrass M. Kaur J. (2025). Vestibular disorders. Prim. Care 52, 15–25. doi: 10.1016/j.pop.2024.09.004 39939085

[B28] Sajjadi H. Paparella M. M. (2008). Meniere's disease. Lancet 372, 406–414. doi: 10.1016/S0140-6736(08)61161-7 18675691

[B29] Sharon J. D. Hullar T. E. (2014). Motion sensitivity and caloric responsiveness in vestibular migraine and Meniere's disease. Laryngoscope 124, 969–973. doi: 10.1002/lary.24285 23818082 PMC3883909

[B30] Smyth D. Britton Z. Murdin L. Arshad Q. Kaski D. (2022). Vestibular migraine treatment: a comprehensive practical review. Brain 145, 3741–3754. doi: 10.1093/brain/awac264 35859353 PMC9679161

[B31] Sun W. Guo P. Ren T. Wang W. (2017). Magnetic resonance imaging of intratympanic gadolinium helps differentiate vestibular migraine from Ménière disease. Laryngoscope 127, 2382–2388. doi: 10.1002/lary.26518 28220492

[B32] Taylor R. L. Zagami A. S. Gibson W. P. Black D. A. Watson S. R. Halmagyi M. G. . (2012). Vestibular evoked myogenic potentials to sound and vibration: characteristics in vestibular migraine that enable separation from Meniere's disease. Cephalalgia 32, 213–225. doi: 10.1177/0333102411434166 22259049

[B33] Velenovsky D. S. (2015). Electronystagmography and videonystagmography (ENG/VNG). Ear Hear. 36:e61. doi: 10.1097/AUD.0000000000000141 25621855

[B34] Villar-Martinez M. D. Goadsby P. J. (2024). Vestibular migraine: an update. Curr. Opin. Neurol. 37, 252–263. doi: 10.1097/WCO.0000000000001257 38619053 PMC11064914

[B35] Wu Q. Dai C. Zhao M. Sha Y. (2016). The correlation between symptoms of definite Meniere's disease and endolymphatic hydrops visualized by magnetic resonance imaging. Laryngoscope 126, 974–979. doi: 10.1002/lary.25576 26333096

[B36] Young A. S. Lechner C. Bradshaw A. P. MacDougall H. G. Black D. A. Halmagyi G. M. . (2019). Capturing acute vertigo: a vestibular event monitor. Neurology 92, e2743–e2753. doi: 10.1212/WNL.0000000000007644 31092626

[B37] Young A. S. Nham B. Bradshaw A. P. Calic Z. Pogson J. M. Gibson W. P. . (2022). Clinical, oculographic and vestibular test characteristics of Ménière's disease. J. Neurol. 269, 1927–1944. doi: 10.1007/s00415-021-10699-z 34420063

[B38] Zhai Q. Chen Q. Zhang N. Li H. Yu Q. Pan Y. . (2025). Exploring vestibulocerebellum-vestibular nuclei-spinal trigeminal nucleus causals communication and TRPV2 ion channel in a mouse model of vestibular migraine. J. Headache Pain 26:47. doi: 10.1186/s10194-025-01986-5 40045241 PMC11881311

