# Supplementary material for: Differentiating vestibular migraine from Meniere's disease: an analysis of clinical features, videonystagmography, and caloric testing
Source: Front Neurosci. 2026 Jan 13;19:1745820. doi: 10.3389/fnins.2025.1745820 (PMC12835259; doi:10.3389/fnins.2025.1745820)
Supplement: Supplementary file 1 [file Table_1.docx]

**Supplementary Table 1. Clinical characteristics of VM and MD patients included in versus excluded from the caloric test analysis.**

Comparisons were made using Fisher's exact test for binary variables or the chi-square test as appropriate.

***p*<0.01

Abbreviation: VM, vestibular migraine; MD, Meniere’s disease; SD, standard deviation; n, number.

| **Variable** | **VM (n=108)** | | | **MD (n=65)** | | |
| --- | --- | --- | --- | --- | --- | --- |
|  | **Included**  **(n=67)** | **Excluded**  **(n=41)** | ***P* value** | **Included**  **(n=54)** | **Excluded**  **(n=11)** | ***P* value** |
| Gender, male, n (%) | 12, (17.9) | 4, (9.8) | 0.247 | 27, (50.0) | 8, (7.3) | 0.168 |
| Age, mean (SD), years | 60.4 (13.0) | 68.3 (10.8) | 0.006** | 61.7 (12.3) | 55.1 (15.0) | 0.800 |
| History of headache, n (%) | 34, (50.7) | 22, (53.7) | 0.769 | 2, (3.7) | 3, (27.3) | 0.007** |
| Characteristics of dizziness |  |  |  |  |  |  |
| Vertigo, n (%) | 56, (83.6) | 36, (87.8) | 0.781 | 51, (94.4) | 11, (100) | 1.000 |
| Rocking, n (%) | 22, (32.8) | 12, (29.3) | 0.831 | 26, (48.1) | 3, (27.3) | 0.517 |
| Light-headed, n (%) | 23, (34.3) | 12, (29.3) | 0.674 | 9, (16.7) | 1, (9.1) | 1.000 |
